# Two Coarse Spatial Patterns of Altered Brain Microstructure Predict Post-traumatic Amnesia in the Subacute Stage of Severe Traumatic Brain Injury

**DOI:** 10.3389/fneur.2020.00800

**Published:** 2020-09-04

**Authors:** Sara H. Andreasen, Kasper W. Andersen, Virginia Conde, Tim B. Dyrby, Oula Puonti, Lars P. Kammersgaard, Camilla G. Madsen, Kristoffer H. Madsen, Ingrid Poulsen, Hartwig R. Siebner

**Affiliations:** ^1^Danish Research Centre for Magnetic Resonance (DRCMR), Centre for Functional and Diagnostic Imaging Research, Copenhagen University Hospital Hvidovre, Hvidovre, Denmark; ^2^Research Unit on Brain Injury Rehabilitation Copenhagen (RUBRIC), Department of Neurorehabilitation, Traumatic Brain Injury, Copenhagen University Hospital Rigshospitalet, Copenhagen, Denmark; ^3^Mental Health Services East, Psychiatry Region Zealand, Roskilde, Denmark; ^4^Clinical Neuroscience Laboratory, Department of Psychology, Norwegian University of Science and Technology, Trondheim, Norway; ^5^Department of Applied Mathematics and Computer Science, Technical University of Denmark, Lyngby, Denmark; ^6^Department of Neurology, Copenhagen University Hospital Rigshospitalet, Copenhagen, Denmark; ^7^Department for Radiology, Centre for Functional and Diagnostic Imaging Research, Copenhagen University Hospital Hvidovre, Hvidovre, Denmark; ^8^Research Unit Nursing and Health Care, Health, Aarhus University, Aarhus, Denmark; ^9^Institute of Clinical Medicine, Faculty of Health and Medical Sciences, University of Copenhagen, Copenhagen, Denmark; ^10^Department for Neurology, Copenhagen University Hospital Bispebjerg, Copenhagen, Denmark

**Keywords:** diffusion tensor imaging, traumatic brain injury, partial least squares analysis, post-traumatic amnesia, prediction, disorders of consciousness

## Abstract

**Introduction:** Diffuse traumatic axonal injury (TAI) is one of the key mechanisms leading to impaired consciousness after severe traumatic brain injury (TBI). In addition, preferential regional expression of TAI in the brain may also influence clinical outcome.

**Aim:** We addressed the question whether the regional expression of microstructural changes as revealed by whole-brain diffusion tensor imaging (DTI) in the subacute stage after severe TBI may predict the duration of post-traumatic amnesia (PTA).

**Method:** Fourteen patients underwent whole-brain DTI in the subacute stage after severe TBI. Mean fractional anisotropy (FA) and mean diffusivity (MD) were calculated for five bilateral brain regions: fronto-temporal, parieto-occipital, and midsagittal hemispheric white matter, as well as brainstem and basal ganglia. Region-specific calculation of mean FA and MD only considered voxels that showed no tissue damage, using an exclusive mask with all voxels that belonged to local brain lesions or microbleeds. Mean FA or MD of the five brain regions were entered in separate partial least squares (PLS) regression analyses to identify patterns of regional microstructural changes that account for inter-individual variations in PTA.

**Results:** For FA, PLS analysis revealed two spatial patterns that significantly correlated with individual PTA. The lower the mean FA values in all five brain regions, the longer that PTA lasted. A pattern characterized by lower FA values in the deeper brain regions relative to the FA values in the hemispheric regions also correlated with longer PTA. Similar trends were found for MD, but opposite in sign. The spatial FA changes as revealed by PLS components predicted the duration of PTA. Individual PTA duration, as predicted by a leave-one-out cross-validation analysis, correlated with true PTA values (Spearman *r* = 0.68, *p*_permutation_ = 0.008).

**Conclusion:** Two coarse spatial patterns of microstructural damage, indexed as reduction in FA, were relevant to recovery of consciousness after TBI. One pattern expressed was consistent with diffuse microstructural damage across the entire brain. A second pattern was indicative of a preferential damage of deep midline brain structures.

## Introduction

Traumatic brain injury (TBI) is a major health problem with an estimated incidence of 69 million new cases per year worldwide (1). In the US, this translates to a total of 2.87 million emergency department visits, hospitalizations, or deaths in 2014, and relative to numbers from 2006, this was an increase of 53% (cdc.gov/traumaticbraininjury/data). The most impairing outcome second to fatality in severe TBI is disorders of consciousness. Traumatic axonal injury (TAI) is regarded the decisive injury for cognitive functional outcome and occurs when high-velocity accelerative and rotational forces due to the nature of the impact interact with the inertia of the brain, causing abrupt movements inside the skull (2). TAI of the long-range axons interconnecting the arousal network of the brainstem-midbrain, the medial fronto-parietal cortex, and the deep gray matter (GM) nuclei of thalamus and basal ganglia is believed causal of post-traumatic lowered level of consciousness (3, 4), and degree and duration of disordered consciousness is regarded the single most influential symptom monitoring and prognosticating severity of TBI (5).

Survivors of coma gradually progress to successive higher levels of consciousness, a process that can take anywhere from days to months and not infrequent pauses in states of unresponsive wakefulness syndrome/vegetative state (UWS/VS) or the minimally conscious state (6). Monitoring progress through clinical bedside evaluation of patient behavior is often misleading and underrepresenting the true level of consciousness (7). Accurate neuroimaging markers of microstructural changes that can be related to physiological function of brain connections for aiding reliable prognostication are therefore highly desirable.

Diffusion-weighted magnetic resonance imaging (dMRI) has been widely used in TBI research to derive quantitative measures of microstructural change due to TAI and to relate these dMRI-based measures with clinical outcome (8–10). A common way to analyze dMRI data is to fit a 3D ellipsoid, a so-called tensor, to the diffusion signal obtained in each voxel. Tensor-based analysis of dMRI data is referred to as diffusion tensor imaging (DTI) and yields quantitative indices of local diffusivity and tissue anisotropy. Fractional anisotropy (FA) and mean diffusivity (MD) are the most common indices, which are highly sensitive to white matter (WM) changes (11–13). Also, DTI usage in GM pathology is increasingly recognized. Debranching of dendrite trees and interstitial edema are suggested mechanisms of FA and MD increase, respectively, and observed in studies of patients with various GM affection, such as TBI, Alzheimer's disease, and multiple sclerosis (14–19). Yet, FA and MD are not very specific in terms of the underlying microstructural change. While high sensitivity for outcome is achieved on a group level, the low specificity of DTI-based metrics to specific anatomical features such as axonal density and organization challenges the transformation of DTI into a clinically relevant prognostic tool by itself at the individual level (20, 21).

Despite the low specificity, refining DTI methods for individual assessment of the structural network integrity within the mesial and posterior brain territories, encompassing networks of both arousal and awareness (22–24), still poses a promising candidate for biomarkers prognosticating disorders of consciousness after severe TBI. Yet, the complexity of inter-connectiveness and inter-dependence within these networks remains a substantial challenge, and many promising attempts aim for advanced technical solutions, with great acquisition and analytic demands (12, 21, 25–30).

In this study, we adopted a novel and simple way to analyze DTI-based microstructural changes and their relationship to the recovery post-traumatic disorders of consciousness, which also could mitigate the challenges mentioned above, in the implementation of DTI as a clinically applicable prognostic tool in the subacute stage after severe TBI. According to the centripetal model of TBI, the force inflicted during the trauma scales with the depth of shear strain injury in the brain (31–33). In agreement herewith, we partitioned the brain into five bilateral regions enabling detection of global as well as spatially accentuated changes in brain microstructure. Using a partial least square (PLS) regression analysis, we expected to uncover both a pattern of global TAI-induced DTI changes and a pattern of preferential damage in the deep and mesial brain regions relative to the hemispheric brain regions that may have predictive value at the individual level, predicting duration of PTA as a clinical index of TBI severity and prognosis.

We chose a coarse brain parcellation that divided the brain into five regions based on two considerations. First, the parcellation should be capable of capturing differences in TBI-induced microstructure in deep vs. superficial brain regions. This was based on the hypothesis that a stronger involvement of deep brain structures would be indicative of a stronger traumatic impact on the brain affecting the deep brainstem and thalamic structures and thus be predictive of PTA duration. Second, this parcellation method was clinically applicable and feasible in a patient population with severely injured brains and disturbed normal anatomy, in which advanced brain segmentation often fails.

## Materials and Methods

This study is part of a larger project described by Conde et al. (34). Patients were recruited from the Department of Neurorehabilitation, Traumatic Brain Injury, Copenhagen University Hospital Rigshospitalet in Denmark at the time of admission in the subacute stage after severe TBI. Inclusion criteria were age above 17 years, closed head injury with instant loss of consciousness (GCS below 9) indicating TAI and followed by coma according to medical records. Patients were excluded if they are not in a state of disordered consciousness at the time of inclusion, had craniectomies, have had major stroke or hemorrhage and substantial brainstem lesions, or had a history of neurological or major psychiatric disease. Twenty-one healthy controls matched by age, sex, and length of educational background were included consecutively for comparative data.

The project was conducted in accordance with the Declaration of Helsinki and approved by the Danish Capital Region Committee on Health Research (H-4-2013-186) and the Danish Data Protection Authority (no 2007-58-0015). Consent for participation was initially given by patient proxy and general practitioner, and if the patient emerged to a state of full consciousness, informed consent was obtained from the patients themselves.

We screened 143 newly admitted patients from October 2014 till February 2018. Seventeen of the 143 patients fulfilled the inclusion criteria and could be included in the study. In 10 of the 17 cases, TBI was caused by traffic accidents. In three cases, TBI was due to falls, one of which was non-accidental. One patient was found unconscious in his home, showing signs of severe head injury, but no mechanism of injury could be established. Three of the 17 patients who were initially included dropped out or had to be excluded from analyses due to low image quality. Thus, data analysis was based on 14 patients with an age range from 18 to 77 years. The patients' characteristics are listed in Table 1. Twenty-one matched healthy controls (2 females, 19 males; mean age, 41 years; age range, 18–68 years) were used as comparison. Patients and healthy control data are summarized in Table 2. Patients were characterized with very severe TBI (5, 35). Initial Glasgow Coma Scale (GCS) score (36) prior to sedation had a median of 3 with a maximum of 8, the Rotterdam CT-cerebrum score (37) had a median of 2 (the scale range is 0–6), and PTA was on average 190 days (range, 62–365). One patient in UWS/VS died during the trial 173 days post-injury from complications to TBI. The following medical conditions and complications during patients' admission to rehabilitation were recorded: Infections: pneumonia (*n* = 6), urinary tract infection (*n* = 9); Cardiovascular: venous thromboembolic (*n* = 2); Neurological/neuroendocrine: paroxysmal sympathetic hyperactivity (*n* = 4), hydrocephalus (*n* = 1), seizures (*n* = 2), and neuroendocrine dysfunction (*n* = 1); Immobility: pressure ulcers (*n* = 2) and spasticity (*n* = 7); Psychiatric: psychosis (*n* = 1) and agitation (*n* = 2). One patient had received anticoagulant medication prior to injury and all patients either received antiplatelet or anticoagulant medication as preventive anti-thrombotic treatment after injury (*n* = 14). Four patients were prone to non-traumatic microbleeds prior to injury because of arterial hypertension (*n* = 3) or atrial fibrillation (*n* = 1). The patient demographics and clinical variables are reproduced from paper of the same authors (38).

**Table 1:** Patients' characteristics.

**Patient**	**Trauma mechanism**	**CTc - focal brain injuries**	**Initial GCS score**	**ISS score**	**Duration of mechanical ventilation (days)**	**GOSE 1Y after injury**	**PTA**
01	Traffic accident		4	24	12	6	76
02	Fall	SAH, SDH, EDH, MLS	3	35	33	3	221
03	Uncertain	SDH, MLS	6	25	14	4	356
04	Traffic accident	SAH, SDH, CON (Bilateral temporal/basofrontal region)	3	30	39	3	365
05	Traffic accident	SAH, IVH, IMI	3	59	25	^*^	^*^
06	Traffic accident	SAH, SDH, CON (Right side temporal region)	3	27	29	7	77
07	Fall	SDH, CON (Left side frontal region)	3	16	20	3	199
08	Fall	SAH, SDH	8	31	29	4	62
09	Traffic accident	SAH, EDH, IVH, CON (Left side temporal region), IMI	4	33	20	4	104
10	Traffic accident	SDH, IVH	3	34	16	4	330
11	Traffic accident	SDH, IVH	6	27	27	4	286
12	Traffic accident	SAH, SDH, IVH, CON (Basofrontal region)	3	43	15	6	102
13	Traffic accident	SAH, SDH, EDH	7	24	13	3	86
14	Traffic accident	EDH, SAH, CON (Bilateral rostral parietal region)	3	43	18	6	212
Mean/median			3 [3–8] (median and range)	31 [16–59] (median and range)	22 (8) (mean and SD)	4 [3–7] (median and range)	190 (114) (mean and SD)

*1Y, one year after trauma; CON, contusion with location noted in brackets; CTc, CT of cerebrum after initial trauma; EDH, epidural hemorrhage; GCS, Glasgow Coma Scale; GOSE, Glasgow Outcome Scale Extended; ISS, Injury Severity Scale; IVH, intraventricular hemorrhage; IMI, intermediary injury (affecting the basal ganglia and/or thalamus); MLS, midline shift; PTA, post-traumatic amnesia; SAH, subarachnoid hemorrhage; SDH, subdural hemorrhage*.

* *Indicates missing data. This patient died 173 days after TBI*.

**Table 2:** Summary of patients' and healthy controls' characteristics.

**Variable**	**Patients included (*N* = 14)**	**Healthy controls (*N* = 21)**
Age (years)	44 (18–77)	41 (18–68)
Female/male	2/12	2/19
Unskilled/skilled/academic career	4/8/2	3/15/3
Glasgow Coma Scale at Injury (3–15)	3 (3–8)	_
Rotterdam Severity Score (1–6)	2 (1–4)	_
Injury Severity Score (1–75)	31 (16–59)	_
Days in mechanical ventilation	22 (12–39)	_
Post-traumatic amnesia (days)	190 (62–365)	_
Time from injury to scan (days)	44 (26–81)	_
Coma Recovery Scale-revised (0–23)	12 (1–23)	_
Early Functional Abilities (20–100)	39 (29–94)	_
Functional Independence Measure (18–126)	18 (18–84)	_

*To anonymize the individual patient information, sex and age are not reported. Sex and level of educational background are reported as ratios. Glasgow Coma Scale, Rotterdam CT score and Injury severity score are reported as median and range. Age, days in mechanical ventilation, post-traumatic amnesia, and days from trauma to scan are reported as mean and range. Coma Recovery Scale-revised, Early Functional Abilities, and Functional Independence Measure are all measured at the time of the scanning and reported as median and range*.

### Magnetic Resonance Imaging (MRI)

Whole-brain MRI scans were acquired on a 3-T whole-body MR system (Verio, Siemens Medical Systems, Germany) using a 32-channel head coil. The protocol included 3D T1-weighted (T1w) Magnetization Prepared Rapid Gradient Echo images [MPRAGE—repetition time (TR) = 1900 ms; echo time (TE) = 2.23 ms; field of view (FoV) = 250 × 250 mm; slab thickness = 176 mm], 3D T2-weighted Sampling Perfection with Application Optimized Contrast using different flip angle Evolutions images (SPACE—TR = 3200 ms; TE = 409 ms; FoV = 250 × 250 mm, slab thickness = 176 mm), and 3D Fluid Attenuation Inversion Recovery images (FLAIR—TR = 5000 ms; TE = 395 ms; FoV = 250 × 250 mm; slab thickness = 160 mm). All of the sequences were acquired in 1-mm^3^ isotropic resolution. Susceptibility Weighted Imaging (SWI) (TR = 28 ms; TE = 20.0 ms; FoV = 230 mm × 173 mm) was acquired in a resolution of 0.8 × 0.7 × 1.2 mm^3^ and diffusion-weighted imaging with 10 b = 0 s/mm^2^ and b = 1,000 s/mm^2^ in 62 directions (TR = 11,060 ms; TE = 78 ms) was acquired in 2.3-mm^3^ isotropic resolution.

All patients were scanned twice as a part of the parent project (34). In the DTI analyses, we used the initial scan acquired within 2 weeks after admission to rehabilitation, except in two cases due to patients' motions and thus low image quality. In these two cases, we used the second scan acquired 6–10 weeks after admission. Likewise, motion corruption resulted in inferior image quality in seven cases of the initial SWI and accordingly traumatic microbleeds were outlines from the second scan. The time of scan relative to the TBI is denoted by the scan used for the DTI analyses and represented in Table 1.

**Figure 1:**
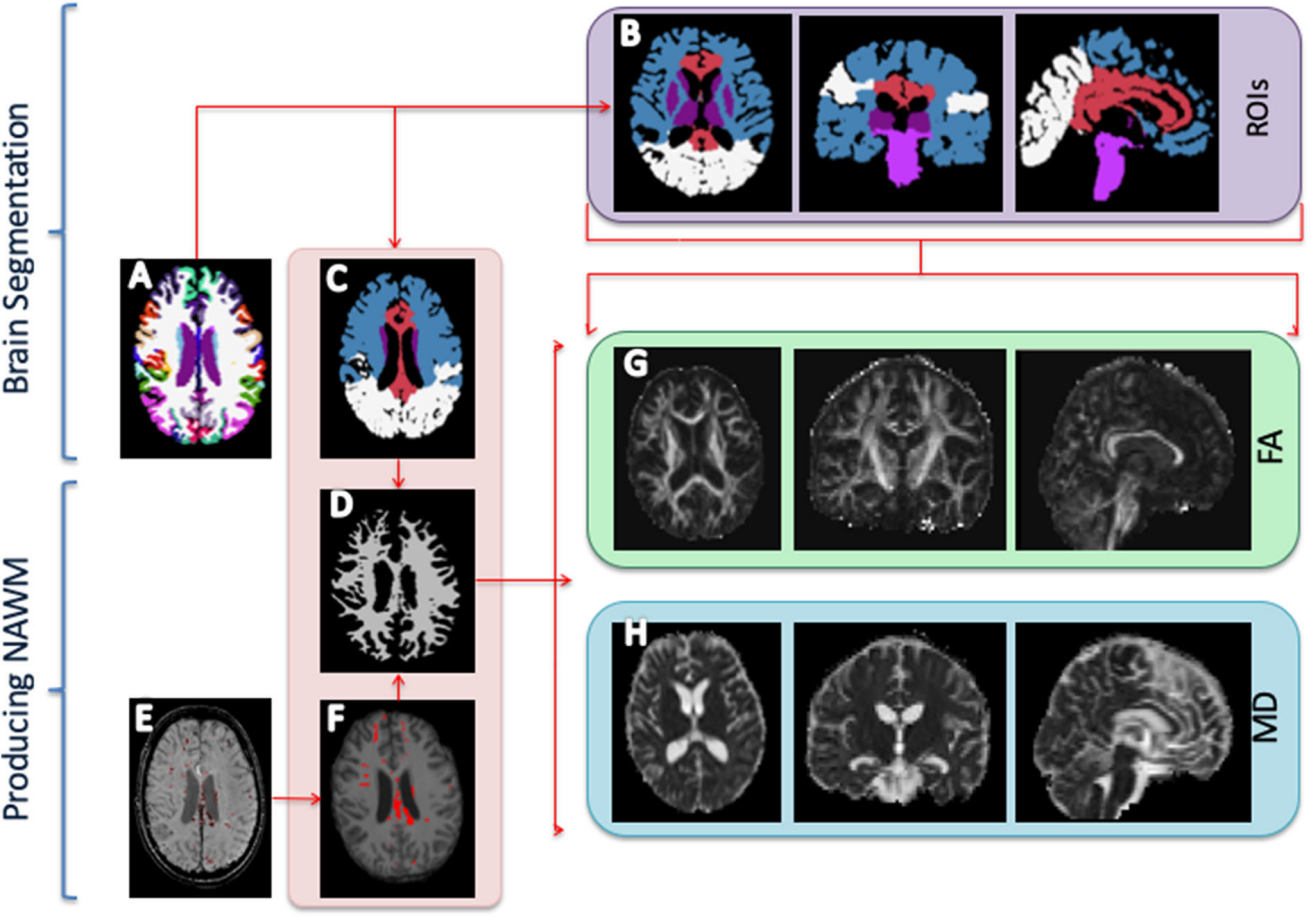
Image processing. **(A)** Freesurfer segmentation. **(B)** Regions of interest (ROIs) segmentation: Fronto-temporal region (blue); parieto-occipital region (white); corpus callosum and parasagittal white matter (red); brainstem (pons, midbrain, and medulla) (dark purple); basal ganglia (caudate, putamen, and pallidum); and thalamus (bright purple). **(C)** The ROIs are used to extract regional diffusion tensor imaging indices, i.e., fractional anisotropy (FA) and mean diffusion (MD). **(D)** Normal-appearing white matter. **(E)** Manual outline of mass lesions and traumatic micro bleeds. **(F)** Mask of outlined lesions. **(G)** FA map. **(H)** MD map.

The structural MRI scans (T1w, T2w, FLAIR, and SWI) were co-registered with SPM-12 software (rev. 6470, https://www.fil.ion.ucl.ac.uk/spm/software/spm12). The T1w scans were processed with Freesurfer software (version 6.0, https://surfer.nmr.mgh.harvard.edu) *recon-all* pipeline to produce volume segmentations of the neuroanatomical structures and a surface parcellation of the cortex (Figure 1A). Each subject was manually checked for segmentation errors, and the initial Freesurfer results were corrected and the subject was re-processed with the manual corrections. Based on the segmentation and cortical parcellation using Freesurfer's mri_annotation2label pipeline, the individual brain images were partitioned into five lobular regions (Figures 1B,C): a fronto-temporal region (FT); a parieto-occipital region (PO); a midsagittal region (CC), including the corpus callosum, cingular, and subcingular WM; a brainstem region (BS), including the pons, the midbrain, and upper medulla; and deep intrahemispheric nuclei, namely, basal ganglia and thalamus (BT). Each voxel in cerebral WM was assigned to one of the five regions based on the closest-in-distance cortical label.

Traumatic microbleeds were outlined on the SWI images and other focal lesions (e.g., mass lesions and tissue damage due to surgical procedures) were outlined on the FLAIR scans (Figures 1E,F). The outlines were performed by author SHA and supervised by a consultant neuroradiologist and author CGM. Outlines were drawn in JIM Xinapse Systems (http://www.xinapse.com/j-im-8-software/) and masked out of the FSL segmentation to form normal-appearing WM masks (Figure 1D).

The dMRI data were pre-processed using an in-house script, including Gibbs un-ringing (39), motion correction, susceptibility correction, and eddy current correction using the *topup* and *eddy* tool as implemented in the FSL software (https://fsl.fmrib.ox.ac.uk) (40, 41). FA and MD maps were computed with FSL toolbox *dtifit* (Figures 1G,H). Before further analyses, all ROIs were eroded by 1 voxel in all dimensions and the four ROIs of primarily WM (FT, PO, CC, and BS) were further thresholded with FA > 0.3 to reduce partial volume effects. The BT ROI, only composed of GM, was not subjected to thresholding.

### Clinical Outcome Variable

The duration of post-traumatic amnesia (PTA) was chosen as clinical outcome of interest in this study. PTA constitutes the time from TBI until the patient is able to consolidate new information and have day-to-day recollection (5). We chose PTA because PTA is considered the best clinical variable for classification of clinical severity (5, 35, 42, 43). Even though the link between PTA beyond 70 days and clinical outcome has not formally been established to date, a recent review of the literature concluded that PTA can be used as a continuous variable for greatest accuracy in outcome prediction (5). To determine duration of PTA, patients were assessed by a trained neuropsychologist with Galveston Orientation and Amnesia Test (GOAT) (44). A GOAT score above 75 twice, i.e., when the patient is able to recall episodic memory with a 24-h elapse, marked the end of PTA (45). PTA duration in patients, who were still in PTA at the time of discharge from the department (7 of the 14 patients), was based on interviews of and reports from the healthcare professionals at the rehabilitation facility, where the patient was discharged to. In five of the seven patients discharged in PTA, the patients were transferred to a highly specialized rehabilitation facility and followed by a trained neuropsychologist. In the last two patients, the duration of PTA was qualified through reports from non-trained healthcare professionals and patients' relatives. Functional ability was measured by the Early Functional Abilities (EFA) and FIM_TM_ scores. EFA describes clinically observable changes of a patient's early functional abilities. The EFA Scale (46) contains 20 items and assesses early basic abilities related to four functional areas: vegetative, face and oral, sensory-motor, and sensory-cognitive. The scores range from 20 to a maximum of 100 points. High scores indicate better functional ability. The EFA has been further validated in a sample of patients with TBI (47). The FIM_TM_ is an 18-item rating scale assessing activities of daily living: self-care, bowel and bladder management, mobility, communication, cognition, and psychosocial adjustment (48). A total FIM_TM_ score ranges from 18 to 126 points, with higher scores indicating greater independence. The FIM_TM_ Scale has shown to be valid and reliable for measuring functional outcome after TBI (49).

### Statistical Analyses

FA and MD analyses within the TBI group were age-adjusted according to a linear trend found in the HC group within each of the five ROIs. This step accounted for age-related WM changes, expected to interact with DTI measures (50, 51).

We performed a PLS regression analysis to examine whether and how the regional expressions of changes in FA and MD relates to PTA. PLS provides an alternative to standard regression and is suited to investigate multi-dimensional data, for which the effect is expected to be driven by complex interactions of the variables of interest. The model reduces the multivariate data into a number of orthogonal components, which represents the maximal co-variance between input and outcome variable(s) (52). Mean FA and MD for each partitioned brain region were first *z* score transformed across subjects and then entered into the PLS analyses (X matrix) to predict the PTA scores (Y—also *z* score normalized). Since Y consists of a single score, this corresponds to the PLS1 version. We then correlated the first three components against PTA using Spearman rank correlation with one-tailed *p*-values.

In addition to using FA and MD, we performed the same set of analyses with the count and volume of microbleeds (also *z* score normalized) in the same regions [see Andreasen et al. (38) for how the microbleeds were delineated]. To include data of the diseased patient in the analyses, PTA of this patient was arbitrarily set to 400 days. We set the significance level to *p* < 0.05/3 = 0.017, which is corrected for the three comparisons done within each of the modalities.

To further evaluate the predictive value of both FA and MD as well as traumatic microbleed count and volume, we performed the same analyses using a leave-one-out cross-validation analysis (LOOCV). LOOCV is a technique where one case is left out iteratively, while the remaining cases are used to train the model and the left-out case is used for prediction (52, 53) to provide an unbiased estimate of the prediction performance. Both PTA and DTI data were normalized by subtracting off the mean and divided with the standard deviation without the left-out case. When using the PLS model for prediction, the output is in standardized values. When only considering a single variable, analyses revealed that mean FA values best predicted individual PTA. We then computed *post-hoc* PLS LOOCV analyses, in which we used FA in combination with either MD, traumatic microbleed count, or traumatic microbleed volume to test whether including additional information would improve the model performance.

To evaluate how well the prediction model was able to predict the true PTA scores, we correlated the individual PTA scores (*z* score normalized) with the predicted PTA scores using Spearman's rank correlation. To assess the significance level of the prediction, we performed random permutation testing; to this end, 10,000 PLS analyses were performed with random permutations of the PTA scores across subjects to form an empirical null distribution of correlation values between predicted and true PTA scores. The PTA scores reported were normalized because the model serves as validation of the DTI data's correlation to PTA and no individual predictions can be derived.

Using this random permutation test, the empirical *p*-value can be computed as the fraction of permuted correlations that exceeds the true correlation. The prediction used a one-tailed test as negative predictions would not add meaningful information. Analyses were performed with Matlab R2017A (The MathWorks, Inc., Natick, Massachusetts, USA) using the plsregress function, and statistical significance was defined as *p* < 0.05.

To evaluate to which extent clinical parameters influenced the results, we separately correlated key clinical information (initial GCS, time from trauma to MRI scanning, time in mechanical respiration, FIM_TM_, and EFA) to PTA.

## Results

### Group Differences in Microstructure Revealed With DTI

The patient characteristics and between-group comparison of the DTI data have previously been reported (38).

### Prediction of PTA From Microstructure

We performed PLS regressions of PTA using MD, FA, traumatic microbleed volume, and traumatic microbleed count separately, within the five ROIs to expose the pattern of microstructural changes that precipitated recovery of consciousness. We used the first three components for the analyses as they represented the minimum number of components that assured that more than 80% of data variation was included in the calculations on all four variables. FAcomp1–3 accounted for 86% of data variation. MDcomp1–3 accounted for 92% of data variation. MB volume comp1–3 accounted for 96% of data variation. MB count comp1–3 accounted for 96% of data variation.

**Figure 2:**
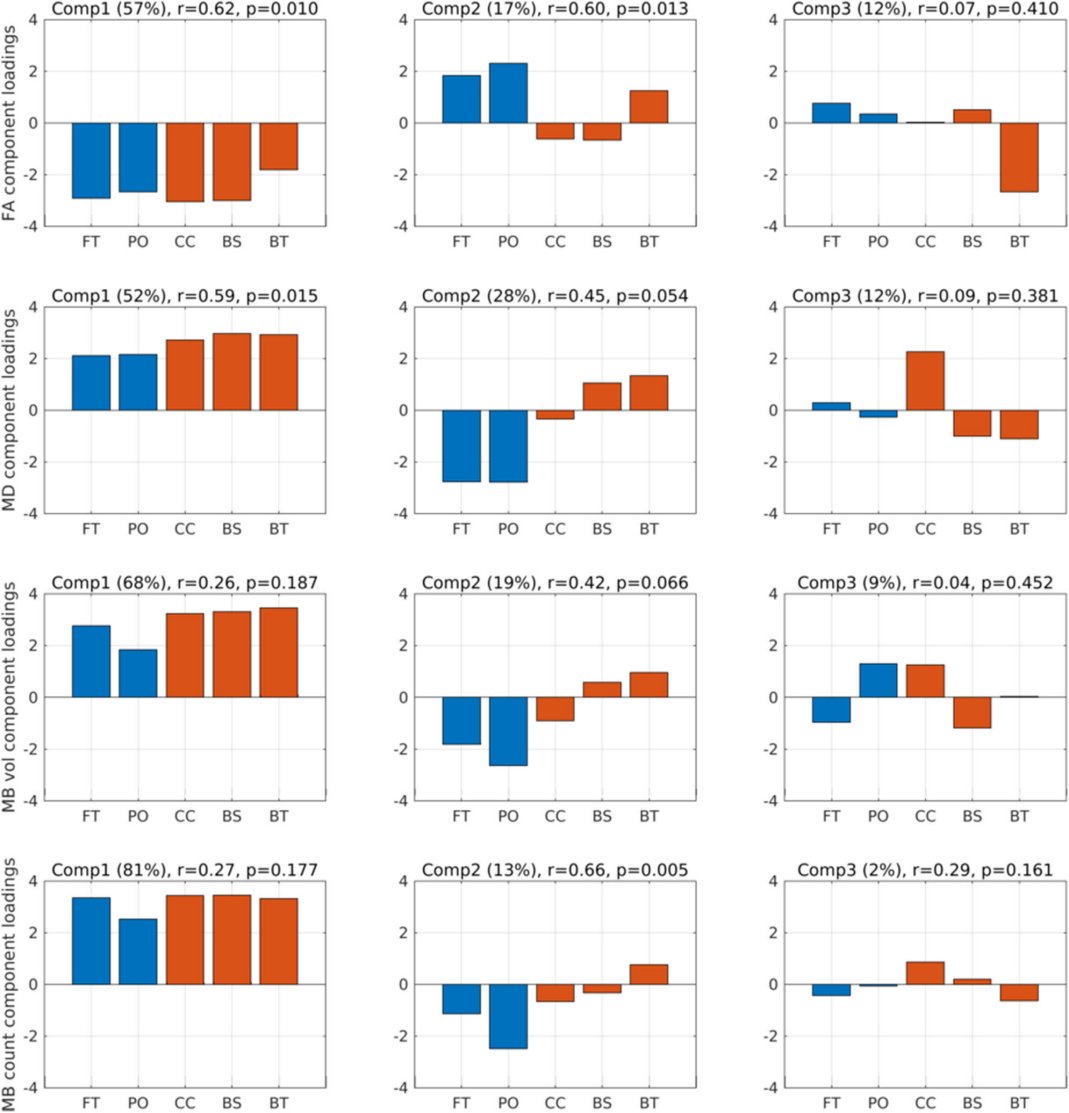
Component loadings. Component loadings (Comp) for the two partial least squares regression analyses predicting post-traumatic amnesia (PTA) using fractional anisotropy (FA) (first row) and mean diffusion (MD) (second row). Above each plot is noted the percentages in parentheses of FA and MD variation explained by the respective components, Spearman *r* correlation and *p* value between the component scores and PTA. Red-colored bars are the deep mesial brain regions, and the blue-colored bars are the hemispheric brain regions. FT, fronto-temporal region; PO, parieto-occipital region; CC, corpus callosum, cingular and subcingular WM; BS, brainstem (pons, midbrain, and medulla); BT, basal ganglia (caudate, putamen, and pallidum) and thalamus.

Figure 2 (top row) shows the component loadings for the FA PLS analyses for the three first components. The loadings show the contribution of each of the regions for that component. The two first components showed significant correlations with PTA scores (FA_COMP1_: Spearman *r* = 0.62, *p* = 0.010; FA_COMP2_: *r* = 0.60, *p* = 0.013), whereas the third component was not significantly correlated to PTA (FA_COMP3_: *r* = 0.07, *p* = 0.41). The loadings on the first component were negative for all five regions, meaning that a general decrease in FA across the five regions were associated with longer PTA duration (i.e., worse outcome). The second component loaded positively on the FT, PO, and BT regions, while negative on the CC and BS regions, suggesting that decreased FA in the deep WM regions relative to FA values in the hemispheric regions resulted in longer PTA duration.

When performing the same PLS analyses based on MD in each of the five brain regions (Figure 2, second row), only component 1 was significantly related to PTA (MD_COMP1_: *r* = 0.59, *p* = 0.015), the second component was only significant at the trend level (MD_COMP2_: *r* = 0.45, *p* = 0.054), and the third component was not significant (MD_COMP3_: *r* = 0.09, *p* = 0.381). MD_COMP1_ loads positive on all five regions, suggesting that a general higher MD value correlates positively with PTA. MD_COMP1_ is basically the same as the inverse of FA_COMP1_, which is also what should be expected since higher MD is usually associated with lower FA. The second MD component, which relates to PTA only at a trend level, suggests that higher mean MD values in BS and BT along with low MD values in the FT and PO region is associated with longer PTA.

As a complementary analysis, we also considered the volume of traumatic microbleeds and count of traumatic microbleeds (Figure 2). PLS of traumatic microbleed_COUNT−COMP2_ identified one spatial component that scaled linearly with PTA duration (Figure 2, fourth row, second component). The second component (traumatic microbleed_COUNT−COMP2_) showed a positive linear relation with PTA (*r* = 0.66, *p* = 0.005). The regional load of this component indicated that traumatic microbleed_COUNT−COMP2_ has a spatial pattern that is characterized by a relative decrease in regional traumatic microbleed count in the cortical regions and a relative increase in the deeper midline regions, especially in basal ganglia and thalamus.

### Predicting PTA Using a Cross-Validation Model

**Figure 3:**
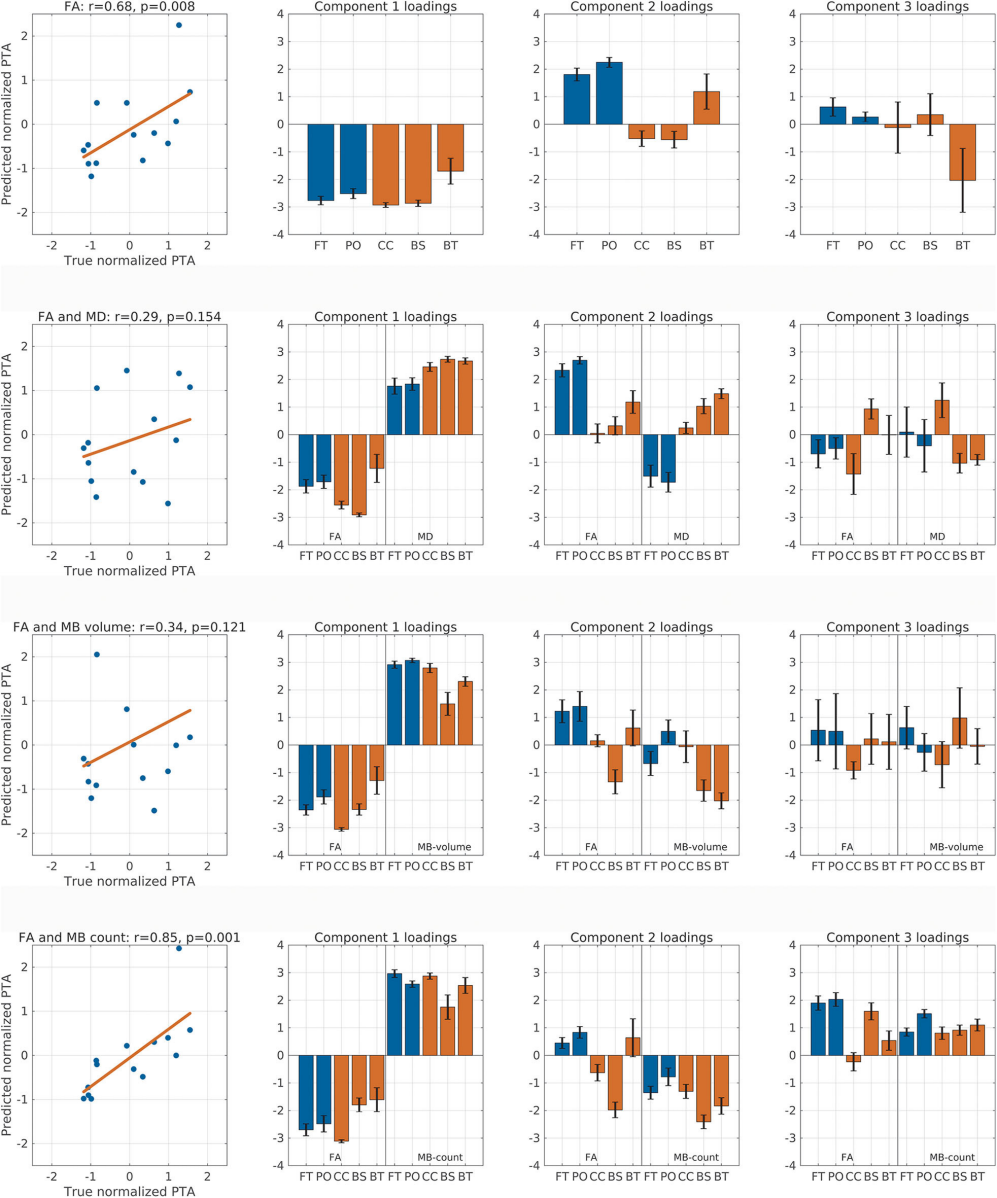
Correlation between predicted and true post-traumatic amnesia (PTA) scores. This figure shows the correlation between predicted and true *z* score normalized PTA scores using partial least square regression with leave-one-out cross-validation. The rows represent analyses performed with fractional anisotropy (FA), FA and mean diffusion (MD), FA and micro bleed (MB) volume, and FA and MB count, respectively. The first column shows the correlation between true and predicted PTA scores and lists the Spearman correlation value as well as the permutation test *p*-value. Columns 2–4 show the component loadings for components 1–3, respectively. Red colored bars are the deep mesial brain regions, and the blue colored bars are the hemispheric brain regions. BS, brainstem (pons, midbrain, and medulla); BT, basal ganglia (caudate, putamen, and pallidum) and thalamus; CC, corpus callosum, cingular and subcingular white matter; FT, fronto-temporal region; PO, parieto-occipital region.

Since our analyses showed a correlation between DTI and traumatic microbleed indices and PTA duration, we tested whether we were able to predict each patient's PTA scores using the spatial expression of FA alone or in combination with either MD, traumatic microbleed count, or traumatic microbleed volume. Therefore, we performed PLS LOOCV analyses using the first three components for each of the analyses (Figure 3). Running the prediction model using the spatial profile of mean FA alone resulted in a significant correlation between predicted and true PTA scores (Spearman *r* = 0.68, *p*_permutation_ = 0.008, Figure 3, row 1). In addition to the scatterplot between predicted and true PTA (first column), the figure also shows the mean (with standard error bars) component loadings for components 1–3 (columns 2–4). For the analysis with FA alone, the loadings are consistent with the loadings from the PLS with all subjects included (Figure 2).

We performed additional exploratory analyses to test whether the addition of any of the other measures would improve the predictive performance when combined with FA. Therefore, we ran the analysis with FA combined with MD (Figure 3, row 2). This analysis did not result in significant correlation between true and predicted PTA duration (Spearman *r* = 0.29, *p*_permutation_ = 0.154). Again, combining FA and traumatic microbleed volume did not result in significant correlation between predicted and true PTA (Figure 3, row 3).

The combination of FA with traumatic microbleed count (Figure 3, row 4) resulted in a higher correlation coefficient (Spearman *r* = 0.85, *p* = 0.001) as compared with the analysis using FA alone. This finding suggests but does not proves that the spatial distribution of microbleeds in the five brain partitions might add additional information about PTA duration when combined with FA. For this analysis, the loadings for component 1 show a general decrease of FA and general increase in traumatic microbleed count will result in longer PTA duration. For component 2, the loadings of the FA variables match the loadings using FA alone. The loadings of the traumatic microbleed count variables, however, are negative for all regions.

We also performed an exploratory analysis based on the MD metric, traumatic microbleed count, and traumatic microbleed volume alone. For MD, there was a trend-wise correlation between predicted and true PTA scores (*r* = 0.45, *p*_permutation_ = 0.057). Using traumatic microbleed counts (*r* = 0.31, *p*_permutation_ = 0.135) or traumatic microbleed volume (*r* = 0.06, *p*_permutation_ = 0.417) alone did not result in significant correlation between predicted and true PTA scores.

As displayed in Table 3, none of the clinical information expected to partially explain the results correlated significantly to PTA. This result suggests that the MRI variables investigated provide valuable information on the PTA duration beyond what could be derived from clinical parameters alone.

**Table 3:** Correlation between clinical variables and outcome parameters.

	PTA^**^	
	**Spearman rho**	***p* value**
Initial GCS^*^	−0.3884	0.1897
Time from trauma to MRI	−0.0644	0.8344
Time in mechanical ventilation	0.2219	0.4662
Functional Independence Measure	−0.2151	0.4804
Early Functional Abilities score	−0.2872	0.3413

* Glasgow Coma Scale;

** *Post-traumatic amnesia. All correlations are corrected for age*.

## Discussion

Our results significantly extend previous work on the use of DTI-based microstructural brain imaging to predict clinical outcome in the subacute stage of severe TBI. Partitioning the brain in five regions, PLS based on mean regional FA values identified two spatial patterns that correlated with individual PTA. These findings suggest that both a diffuse microstructural damage across the entire brain and a preferential damage of deep brain structures are associated to the duration of PTA after severe TBI.

The first pattern that predicted the duration of PTA was a global microstructural change in all five brain regions, reflecting overall TAI burden. The lower the mean FA values and opposing higher MD values in all five brain regions, the longer was the duration of the patient's anterograde amnesia. We attribute reductions of mean FA and increase of MD in WM regions to axonal degeneration of some tract systems caused by TAI. This contrasting pattern of low FA and high MD associated with TAI and WM atrophy is reproduced in multiple studies of patients in the subacute phase of TBI (17, 54–59). Traumatic axonal injury reduces the directional bias of regional water diffusion in the white matter, causing a reduction in regional FA. At the same time, axonal injury leads to an increase of MD in the white matter, because water diffusion is now less restricted.

Of note, between-group comparison of regional FA values in basal ganglia and thalamus revealed higher mean FA in TBI patients relative to controls. Yet, the first component derived from pattern analyses indicated that lower (not higher) mean FA values in the BT region and all other brain regions scaled with PTA duration. This apparent discrepancy points to both decreased and increased FA values in GM that can be explained by the combination of two mechanisms: Firstly, TBI may increase intranuclear FA through microstructural damage of dendritic arborization of intranuclear cells, which increases directed diffusion along internuclear fiber bundles. Secondly, decreased FA values can stem from TAI damage of the internuclear fiber bundles arising from surrounding WM and subsequent Wallerian degeneration of the axonal segments within the deep GM nuclei. Such axonal degeneration of more specific tracts can lead to widespread changes in WM and GM and in crossing fiber regions where one tract being degenerated can even lead to a regional increase in FA as when comparing the FA with the novel microscopic fractional anisotropy (microFA) metric which is unaffected by regional variations in orientation dispersion (60). Thus, a mixture of trauma-related mechanisms that have opposite effects on FA in thalamus and basal ganglia may also explain why FA is less sensitive to tissue damage in deep GM nuclei than MD (61).

In addition to the first global pattern, the PLS regression analyses identified a second spatial component that was predictive of PTA duration. The spatial pattern of the second component is characterized by decreased FA values in deeper WM brain regions relative to the FA values in the superficial hemispheric regions. Hence, the positive predictive value of the second component indicates that a preferential microstructural tissue damage of deep midline structures is associated with a more severe impairment of consciousness and, thus, longer PTA.

DTI-derived metrics are influenced by many biological factors from microstructure at the cellular level to crossing fibers, resulting in a high sensitivity and low specificity to trauma-induced microstructural alterations. Because of the low specificity of DTI-derived microstructural metrics, we cannot infer that a widespread decrease in FA exclusively reflects a loss in axonal integrity. A complex microstructural environment, including non-straight axons, extracellular matrix, and extra-neuronal cells, contributes to DTI metrics, such as FA and MD (62–64). As brain trauma enrolls a cascade of immunological responses, involving protein deposition, morphological changes of glia cells, and edema (64, 65), the extra-axonal contribution to directionality of the diffusion tensor may be even greater than in healthy tissue. Likewise, the complex pathological dynamics of TBI involve concurrent repairing as well as secondary tissue damage (56, 66). These inherent ambiguities complicate the interpretation of DTI indices further. Poor specificity of DTI-based microstructural metrics is outbalanced by the high sensitivity of DTI to microstructural change. The high sensitivity of dMRI-based indices to detect microstructural change explains why the spatial patterns of FA changes contain prognostic information in terms of PTA duration.

The significant linear relationship between the expression of the second spatial FA component and PTA is in good agreement with the centripetal theory or the Ommaya–Gennarelli hypothesis. The theory states that the tensile axonal stress progresses from the outer layers to the core of the brain, (33, 67). The theory is supported by empirical evidence in more than 400 closed head autopsies and histology examinations (68). The centripetal model explains not only the physical effect from inertia of the brain exposed to high velocity acceleration–deceleration and rotation, it also reflects the hierarchical order of networks needed to maintain consciousness.

At the most basic level of consciousness generation is the ascending reticular activation system (ARAS) sustaining arousal and diurnal rhythm. ARAS is known to be densely connected to regulation of basic vegetative functions such as thermo-regulation and the peripheral autonomic nervous system (69, 70). TAI lesions in the brainstem–midbrain region therefore affect not only consciousness but also the entire vegetative homeostasis and thereby the changes of long-term survival in TBI patients. This can be exemplified by a study from Patrick et al. in which lesions located in the brainstem correlated with clinical outcome. They dichotomized outcome of post-traumatic encephalopathy in children suffering from very severe TBI and found that 93% of the children with brainstem lesions were in the unfavorable outcome group with protracted symptom duration (71). Likewise, Mannion et al. found that 11 out of 13 patients with brainstem lesions, in an adult population of severe TBI patients, had unfavorable outcome of death, UWS, or severe disability, whereas only 18 of 33 patients without brainstem lesions had an unfavorable outcome (72). DTI changes in the sub-tentorial part of the brain and mesial midbrain therefore constitutes not only a signature of global TAI burden, according to the Ommaya–Gennarelli hypothesis, but additionally an indication of damage to WM of consciousness supporting networks.

Studies documenting DTI abnormalities in the brainstem related to adverse outcome in TBI are relatively sparse compared to the major sites of common WM susceptibility to shear strain injury (8). The latter could be due to the high morbidity of patients in the post-traumatic coma stage and hence the selected population that proceeds to the subacute phase, in which majority of studies are conducted, has a relatively low degree of WM damage in the sub-tentorial brain (8). Therefore, our finding of a positive association between deep microstructural WM injury measured in the subacute stage of trauma and PTA may predominantly be attributed to traumatic de-afferentiation of deep GM nuclei. Damage to ascending connections from ARAS and spinal afferent somatosensorial input to the thalamic nuclei are presumed to be the background mechanism of reduced net excitatory drive of the basal forebrain observed in disordered levels of consciousness after TBI (4, 73–76). The theory has been substantiated in several studies, including the study of Newcombe et al. showing DTI metrics of deep WM and GM that correctly classified mild from moderate TBI (17), and a significant correlation between cortico-cortical and thalamo-cortical connectivity and level consciousness was reported in two studies of patients with post-traumatic disorders of consciousness (76, 77).

Using the DTI-derived components in our LOOCV-predicted PTA only in the FA model, FA was indicated to be a more reliable indexation of TAI-induced microstructural damage, which is in line with results of previous studies (8). Considering both mean FA and MB load, PLS analysis based on combined DTI/SWI results yielded the highest predictive ability among the metrics tested. Although we were not able to formally test this, our results suggest that the two modalities, DTI and SWI, provide complimentary information about post-traumatic microstructural brain damage. TBI results in a complex microvascular pathology that cannot be accounted for by primary shear strain injury (78). Hence, it is plausible to assume that the combination of DTI and SWI offers a more holistic picture that may increase the ability of structural MRI in the subacute post-TBI stage to predict PTA duration. However, the inclusion of two MRI modalities renders a neurobiological interpretation more complicated. The first component of the PLS results showed that lower loads on FA and higher loads on MB was associated with longer PTA duration. This indicates that microstructural damage caused by TAI and reflected by FA as well as the expression of microbleeds in the entire brain are associated with longer PTA duration. The other two components are more complicated to interpret. Similar to component 1, higher positive loadings on the MB metric were found in component 3. This component suggests that more widespread microbleed burden has an adverse effect on PTA duration, when paired with relatively strong microstructural damage in the pericallosal hemispheric midline region, as indexed by a low mean FA value in the CC ROI. Component 2 is mainly characterized by a relative FA decrease in the brainstem and it seems that a preferential damage of the brainstem region is of prognostic relevance for PTA duration, if the brain has a low microbleed load.

## Limitations

Our study has several limitations. The main limitation is the small sample size. In this study population of very severe TBI, the clinical condition of the individual patient was a substantial limitation in the inclusion of enough patients, as many were unfit for MRI scanning at the time of admission and many had contraindications for MRI, due to surgical procedures. The cross-sectional design precluded long-term assessment of how the microstructural alterations dynamically change over time. Important information on regional pathological development or repair, as well as optimal timing of DTI assessment related to prognosis, would be obtainable in a longitudinal study with repeated measurements. Furthermore, the limited data of 14 patients did not allow inclusion of clinical parameters expected to interact with our results into the prediction model. Future studies of larger patient populations could allow both subgroup analyses and combinations of patient's demographics, clinical variables of relevance, and MRI biomarkers for individual and accurately prognostic models (79–81). The insignificant results of our complementary correlation analyses between key clinical information and PTA suggest that MRI variables, investigated to a large extent, provide independent information on the duration of PTA.

One technical limitation is due to the abnormal brain anatomy inherent in a group of patients after extremely severe TBI. Conventional tissue classification strategies are well known to fail, and patient's brain images are unfit for registration to atlases (25). We therefore used a surface-based method of brain segmentation, although ROIs of WM tracts would have been preferred for examining recovery of consciousness, which largely can be attributed to mesial brain connectivity. A translational limitation is the use of normalized values in the statistical prediction of PTA against true observed PTA, which is a requisite for many machine learning techniques. An additional limitation is the certainty with which we can assign measures of consciousness (and in turn estimation of PTA) to TAI. Focal lesions, such as bi-thalamic or bi-hemispheric injuries, are well-known to also impact consciousness (82, 83). Finally, systemic infection, pharmacological treatments, and raised intra-cranial pressure are possible confounders estimating TAI-induced disorders of consciousness.

## Conclusion

Despite these limitations, our preliminary results are encouraging. Our spatially coarse DTI-based analysis captured two distinct spatial features of microstructural changes that scale positively with the duration of PTA. Our preliminary results indicate that diffuse microstructural brain damage as well as more focused microstructural damage of central brain structures contribute to longer duration of impaired consciousness. This shows that microstructural alterations can be detected with standard DTI-based metrics in the subacute stage of severe TBI and contain relevant prognostic information. Regional FA, a standard DTI-based metric, is sufficiently sensitive to capture both spatial patterns of microstructural damage and may thus be a useful supplementary MRI modality to assist prognostication in the subacute stage despite of its low specificity to TAI (64). However, the usefulness of our DTI-based approach to predict individual PTA duration needs to be validated in larger longitudinal studies.

## Data Availability Statement

Data used in this paper are available in pseudo-anonymized form and can be shared by request from any qualified investigator after approval of a data transfer agreement by the Danish Data Protection Agency.

## Ethics Statement

The project was conducted in accordance with the Declaration of Helsinki and approved by the Danish Capital Region Committee on Health Research (H-4-2013-186) and the Danish Data Protection Authority (no 2007-58-0015). Consent for participation was initially given by patient proxy and general practitioner, and if the patient emerged to a state of full consciousness, informed consent was obtained from the patients themselves.

## Author Contributions

SA, KA, VC, LK, IP, and HS: design. SA, KA, VC, LK, CM, and IP: data collection. SA, KA, VC, TD, OP, LK, CM, KM, IP, and HS: analyses. SA, KA, IP, and HS: co-writing the paper. All authors contributed to the article and approved the submitted version.

## Conflict of Interest

HS has received honoraria as speaker from Sanofi Genzyme, Denmark and Novartis, Denmark, as consultant from Sanofi Genzyme, Denmark, and as senior editor (NeuroImage) and Editor-in-Chief (NeuroImageClinical) from Elsevier Publishers, Amsterdam, The Netherlands. He has received royalties as book editor from Springer Publishers, Stuttgart, Germany. HS holds a 5-year professorship in precision medicine at the Faculty of Health Sciences and Medicine, University of Copenhagen, which is sponsored by the Lundbeck Foundation (Grant Nr. R186-2015-2138). The remaining authors declare that the research was conducted in the absence of any commercial or financial relationships that could be construed as a potential conflict of interest.

## Abbreviations

FS: Freesurfer
LOOCV: Leave-one-out cross-validation analysis
PLS: Partial least square regression
PTA: Post-traumatic amnesia
RAS: Reticular activation system
TAI: Traumatic axonal injury
UWS: Unresponsive wakefulness syndrome.
